# Supplementary material for: Novel Unspecific Peroxygenase from Truncatella angustata Catalyzes the Synthesis of Bioactive Lipid Mediators
Source: Microorganisms. 2022 Jun 22;10(7):1267. doi: 10.3390/microorganisms10071267 (PMC9322767; doi:10.3390/microorganisms10071267)
Supplement: Supplementary file 1 [file microorganisms-10-01267-s001.zip › microorganisms-1752877-supplementary.pdf]

*Supplemental Material*

# Novel Unspecific Peroxygenase from *Truncatella angustata* Catalyzes the Synthesis of Bioactive Lipid Mediators

**Rosalie König [1,*], Jan Kiebist [1,2], Johannes Kalmbach [1], Robert Herzog [3], Kai-Uwe Schmidtke [1], Harald Kellner [3], René Ullrich [3], Nico Jehmlich [4], Martin Hofrichter [3] and Katrin Scheibner [1,*]**

[1] Institute of Biotechnology, Brandenburg University of Technology Cottbus-Senftenberg, Universitätsplatz 1, 01968 Senftenberg, Germany; jan.kiebist@izi-bb.fraunhofer.de (J.K.); johannes.kalmbach@b-tu.de (J.K.); kai-uwe.schmidtke@b-tu.de (K.-U.S.)
[2] Fraunhofer Institute for Cell Therapy and Immunology, Branch Bioanalytics and Bioprocesses IZI-BB, Am Mühlenberg 13, 14476 Potsdam, Germany
[3] Unit of Environmental Biotechnology, TU Dresden—International Institute Zittau, Markt 23, 02763 Zittau, Germany; robert.herzog1@tu-dresden.de (R.H.); harald.kellner@tu-dresden.de (H.K.); rene.ullrich@tu-dresden.de (R.U.); martin.hofrichter@tu-dresden.de (M.H.)
[4] Department of Molecular Systems Biology, Helmholtz-Centre for Environmental Research, UFZ, Permoserstrasse 15, 04318 Leipzig, Germany; nico.jehmlich@ufz.de
* Correspondence: rosalie.koenig@b-tu.de (R.K.); katrin.scheibner@b-tu.de (K.S.)

**Table of contens**

Cultivation of *Truncatella angustata* and isolation of *Tan*UPO ........ 3
Peptide mapping ........ 7
Protein structure simulation and ligand fitting ........ 9

**Tables & Figures**

**Table S1.** Semisynthetic liquid medium for cultivation of *Truncatella angustata* in order to produce *Tan*UPO ........ 3
**Table S2**. Purification of the UPO produced by *Truncatella angustata* ........ 5
**Table S3.** Apparent kinetic parameters of five homologous UPOs including new *Tan*UPO for the substrates veratryl alcohol (VA) and ABTS ........ 7
**Table S4.** $MS^1$ and $MS^2$ [M-H]- signals of by-products formed by enzymatic conversion of AA ........ 14

**Figure S1.** Time-course of UPO production by *T. angustata* ........ 4
**Figure S2.** FPLC elution profile of ~1,100 U *Tan*UPO using anion exchange chromatography ........ 5
**Figure S3.** FPLC elution profile of ~240 U *Tan*UPO I using size exclusion chromatography ........ 6
**Figure S4.** SDS-PAGE of purified *Tan*UPO. ........ 6
**Figure S5.** Effect of pH and temperature on the conversion of AA to 14,15-EET by *Tan*UPO ........ 7
**Figure S6.** Full MS spectra of EETs and HETEs formed by UPOs ........ 8
**Figure S7.** 14,15-EET reference standard ........ 8
**Figure S8.** 11,12-EET reference standard ........ 9
**Figure S9.** 18-HETE reference standard ........ 9
**Figure S10.** 19-HETE reference standard ........ 10
**Figure S11.** LC-MS spectra ($MS^2$) of product ions of 14,15-EET formed by *Tan*UPO, *Mro*UPO, *Mwe*UPO and *Cgl*UPO ........ 10

**Figure S12.** LC-MS spectra (MS²) of product ions of the di-oxyfunctionalized product formed by *Ta*nUPO, *Mro*UPO and *Mwe*UPO ..... 11

**Figure S13**. LC-MS spectra (MS²) of product ions of 11,12-EET formed by *Cgl*UPO, *Mro*UPO and *Mwe*UPO ..... 11

**Figure S14.** LC-MS spectra (MS²) of product ions of suspected the di-epoxide formed by *Cgl*UPO ..... 12

**Figure S15.** LC-MS spectra (MS²) of product ions of 18-HETE formed by *Aae*UPO and *Cra*UPO ..... 12

**Figure S16.** LC-MS spectra (MS²) of product ions of 19-HETE formed by *Aae*UPO and *Cra*UPO ..... 13

**Figure S17.** Amino acid sequence of the *Tan*UPO protein (GenBank: KAH8203164.1) ..... 16

**Figure S18.** Multiple sequence alignment (MSA) plot with the assumed mature *Tan*UPO protein sequence as query ..... 18

**Figure S19.** pLDDT (predicted local distance difference test) plot for the top 5 ranked *Tan*UPO-output models of ColabFold ..... 18

**Figure S20.** Cartoon representation of predicted *Tan*UPO models (left to right) from rank 1 to rank 5 ..... 19

**Figure S21.** PAE (predicted alignment error) plots of the models of *Tan*UPO ..... 19

**Figure S22.** Structural alignment of *Aae*UPO, *Mro*UPO and *Tan*UPO using the CEAlign algorithm ..... 20

**Figure S23.** Topology of the SAC (substrate access channel) of *Aae*UPO forming helices and loops ..... 21

**Figure S24.** Topology of the SAC (substrate access channel) of *Mro*UPO forming helices and loops ..... 22

**Figure S25.** Topology of the SAC (substrate access channel) of *Tan*UPO forming helices and loops ..... 23

**Figure S26.** Kinetic profile of arachidonic acid (AA) conversion to 14,15-EET by *Tan*UPO. ..... 24

Cultivation of *Truncatella angustata* and isolation of *Tan*UPO

The novel peroxygenase, *Tan*UPO, is an extracellular enzyme isolated from liquid cultures of *Truncatella angustata*. The isolate was collected from a fruiting body of the basidiomycetous fungus *Psathyrella conopilus* (order Agaricales) growing on soil mulched with *Robina pseudoacacia* woodchips (Germany, 02994 Bernsdorf, 51°23′51.1″N 14°01′42.2″E). The identity of the isolate (strain S358) that is deposited in our institute's culture collection was confirmed by means of genome (including ribosomal DNA) sequencing (data not shown). *T. angustata* (phylum Ascomycota, order Xylariales) is a coelomycetous fungus, i.e. it produces conidia within a cavity lined by fungal or host tissue, called conidiomata. It is known as a facultative pathogen and endophyte of various plants, and apparently can colonize larger structures (e.g. fruiting bodies) of other fungi. (https://mycocosm.jgi.doe.gov/Truan1/Truan1.home.html).

The production and isolation of *Tan*UPO was performed as follows: An overgrown malt-extract agar plate (20 g $L^{-1}$ malt extract, 20 g $L^{-1}$ agar) incubated at 24 °C for 10 days, was homogenized in 100 mL sterile 0.9% (w/v) NaCl by a dispersing device (ULTRATURRAX®, IKA®-Works, Staufen). For enzyme production, 500-mL Erlenmeyer flasks filled with 200 mL semisynthetic medium pH 6.5 [62] (Table S 1), were inoculated with 2 mL homogenate and incubated on a rotary shaker at 120 rpm and 24 °C (in total 26 flasks). At intervals of 1-3 days, 2 mL culture liquid was taken and centrifuged (17,000 g, 20 min), and the clear supernatant obtained was used for the determination of pH and peroxidase / peroxygenase activity using the ABTS assay (see 2.4). On the third day of cultivation, 2 mL 2 M NaOH was added to each culture flask to counteract the strong drop in pH to 5 - 4.5. During the entire cultivation period of 15 days, the pH increased to ~8 due to ammonification (formation of $NH_3$/$NH_4OH$) [63-64] of the media components (peptones) by fungal enzymes and has already been observed during the expression of other UPOs [57;59]. When the maximum UPO activity had been reached (Fig. S1), the filtrated culture liquid was frozen to precipitate extracellular polysaccharides. The culture liquid of *T. angustata* S358 was separated from the mycelium by filtration through paper filters (GF8 Whatman, Dassel, Germany), afterwards concentrated by two steps of ultrafiltration against 10 mM sodium acetate buffer (pH 6.5) using two tangential flow cassettes (Sartocon Slice Cassette, Hydrosart, cut-off 10 kDa and MinimateTM TFF Capsule / Pall with Omega membrane, cut-off 10 kDa) successively. The concentrated culture filtrate was adjusted to 60% saturation with ammonium sulfate and centrifuged at 20,000 x g for 30 min. The resulting supernatant was desalted using a 10-kDa cut-off membrane (Sartocon Slice Cassette, Hydrosart).

**Table S1.** Semisynthetic liquid medium for the cultivation of *Truncatella angustata* to produce *Tan*UPO.

| Compound | Final concentration |
|---|---|
| Saccharose | 25 g/L |
| $MgSO_4$ x 7 $H_2O$ | 0.3 g/L |
| $FeSO_4$ x 7 $H_20$ | 0.02 g/L |
| $CaCl_2$ | 0.3 g/L |
| $KH_2PO_4$ | 2.0 g/L |
| *di*-Ammonium tartrate | 13.32 g/L |
| Yeast extract | 0.2 g/L |
| Trace element solution[1] | 10 mL/L |

[1] Corresponds to the trace element solution used in the cultivation of strains of the family Ustilaginaceae family [62].

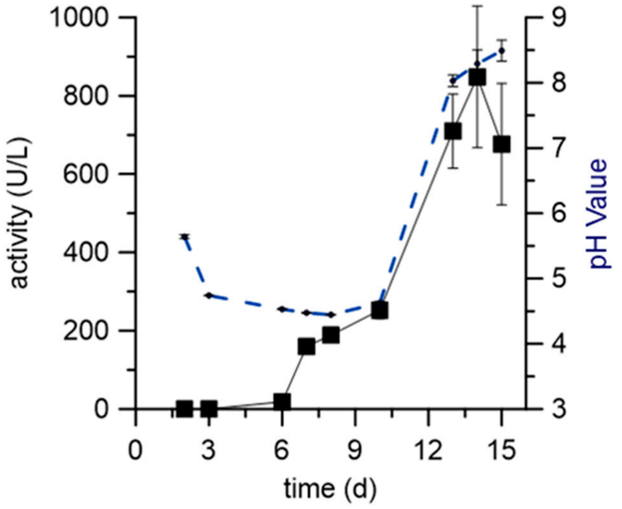


**Figure S1.** Time-course of UPO production (squares) by T. angustata. TanUPO activity was measured with ABTS as substrate at pH 4.5. Data given are means of three culture flasks with standard deviation. The dotted line shows the pH.

The first step of chromatographic purification was anion exchange chromatography (AEC) Q-Sepharose® FF (column AEC XK 50 x 200 mm; GE Healthcare Europe, Freiburg, Germany) using an Äkta Avant system (GE Healthcare Europe, Freiburg, Germany). The sample was loaded onto the column with a sample pump. The column was washed with 10 mM Na acetate (pH 6.5) to remove weakly bound proteins, and then eluted using a linear gradient 0→50% 2 M NaCl in 4 column volumes (CV) at a flow rate of 20 mL $min^{-1}$ and a fraction size of 14 mL (Fig. S2).

After desalting, the *Tan*UPO preparation was applied onto an anion exchanger MonoQ® (column 10 x 100 mm; GE Healthcare) using 10 mM Na acetate (pH 6.9) and an elution gradient of 0→20% 2 M NaCl within 14 CV at a flow rate of 6 ml $min^{-1}$ and a fraction size of 1.5 mL.

Finally, the pooled fractions were further purified via size exclusion chromatography (SEC) on a HiLoad Superdex® 75 pg column (16 x 600 mm; GE Healthcare Europe, Freiburg, Germany). As buffer, 100 mM NaCl in 50 mM Na acetate (pH 6.8) was applied at a flow rate of 1 ml $min^{-1}$ and a fraction size of 1.0 mL (Fig. S3). A protease inhibitor without EDTA (Roche cOmplete™) was added to the collected and combined fractions, and the purified enzyme was stored at -20 °C.

The purification Table S 2 summarizes the different steps of the *Tan*UPO isolation procedure. The purified protein was further analyzed by SDS-PAGE (Fig. S 4). Accordingly, the *Tan*UPO showed two bands in the range of 40-45 kDa. This is due to the presence of two isoforms with MW 40 kDa and 45 kDa. Both isoforms are products of one gene (g2685.t1; theoretical MW 28 kDa) and very probably represent the differently glycosylated protein.

**Table S2.** Purification of the unspecific peroxygenase (TanUPO) secreted by Truncatella angustata.

| **Purification step** | **Total activity (U)** | **Protein amount (mg)** | **Specific activity ($U\ mg^{-1}$)** | **Purifi-cation (fold)** | **Yield (%)** |
|---|---|---|---|---|---|
| Ultrafiltration (cut-off 10 kDa) | 1,128 | 3,288 | 0.3 | - | - |
| $(NH_4)_2SO_4$ precipitation* | 1,094 | 2,310 | 0.5 | 1.4 | 96.9 |
| Q-Sepharose™ FF (AEX) | 691 | 52.5 | 13.2 | 38.4 | 61.3 |
| Mono Q™ fraction I (AEX) | 237 | 14.1 | 16.8 | 49.4 | 21.0 |
| Mono Q™ fraction II (AEX) | 206 | 17.7 | 11.6 | 33.9 | 18.3 |
| Superdex™ 75 pg *Tan*UPO I (SEC) | 155 | 3.1 | 50.0 | 145.9 | 13.8 |
| Superdex™ 75 pg *Tan*UPO II (SEC) | 163 | 4.8 | 33.9 | 98.7 | 14.4 |

* after desalting.

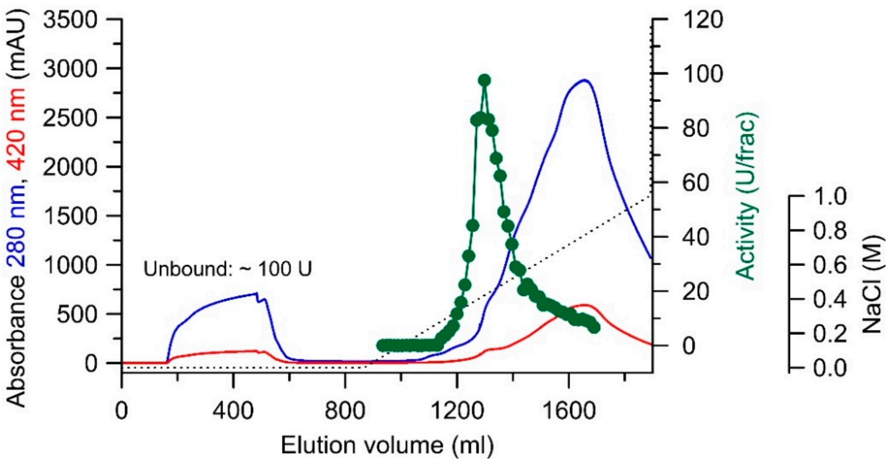


**Figure S2.** FPLC elution profile of *Tan*UPO (~1,100 U). Separation was performed by anion exchange chromatography on Q sepharose® (XP 50/200). Absorption at 280 nm (total protein, blue line), at 420 nm (heme, red line), UPO activity measured with ABTS (•) and NaCl gradient (dotted line).

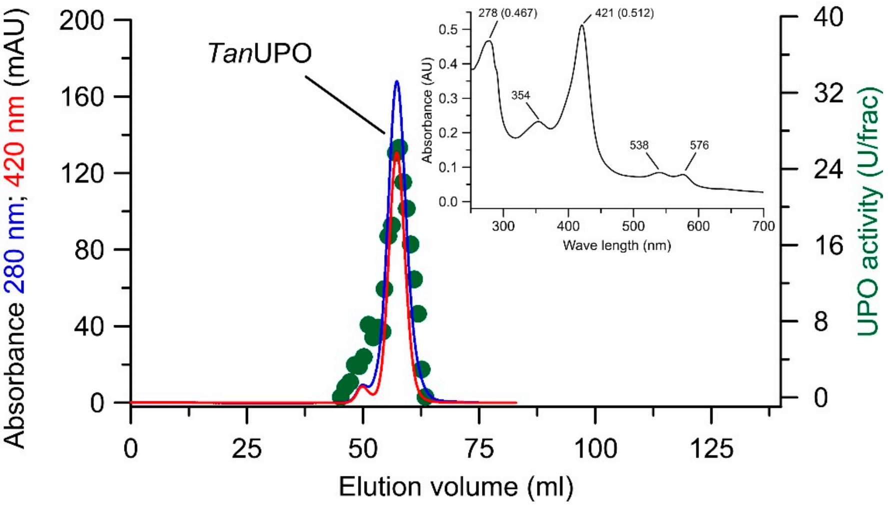


**Figure S3.** FPLC elution profile of TanUPO I (~240 U). Separation was performed using size exclusion chromatog-raphy on Superdex® 75 pg (column 16 x 600 mm). Absorption at 280 nm (blue line), at 420 nm (red line) and UPO activity (ABTS) (•). The inset shows the UV-Vis spectrum of pooled TanUPO I.

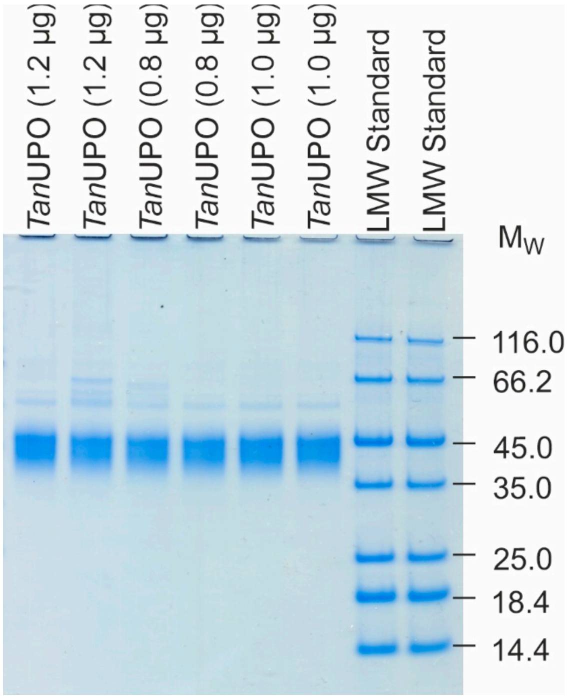


**Figure S4.** SDS-PAGE of purified TanUPO. The 10% NuPAGE SDS gel was stained with the colloidal Blue Staining kit (Invitrogen, Waltham, Massachusetts, USA). LMW standard: Pierce™ unstained protein molecular weight marker #26610 (14.4 – 116.0 kDa) (Thermo Scientific, Waltham, Massachusetts, USA). TanUPO comprises two isoforms with different masses (MW 45 kDa and 40 kDa).

**Table S3.** Apparent kinetic parameters of five homologous UPOs including new *Tan*UPO (first row) for the substrates veratryl alcohol (VA) and ABTS.

| Enzyme | Substrate | $K_m$ (µM) | $k_{cat}$ ($s^{-1}$) | $k_{cat}/K_m$ ($M^{-1}$ $s^{-1}$) | Reference |
|---|---|---|---|---|---|
| *Tan*UP | ABTS[a] | 257 | 31 | $1.21 \cdot 10^5$ | |
| O | VA[b] | 261 | 3 | $1.15 \cdot 10^4$ | |
| *Cgl*UPO | ABTS[a] | 106 | 111 | $1.04 \cdot 10^6$ | |
| | VA[b] | 346 | 19 | $5.49 \cdot 10^5$ | [60] |
| *Mro*UP | ABTS[a] | 71 | 25 | $3,53 \cdot 10^5$ | |
| O | VA[c] | 279 | 49 | $1.76 \cdot 10^5$ | [59] |
| *Aae*UP | ABTS[a] | 37 | 283 | $7.67 \cdot 10^6$ | |
| O | VA[b] | 2,367 | 85 | $3.58 \cdot 10^4$ | [57] |
| *Cra*UPO | ABTS[a] | 49 | 123 | $2.51 \cdot 10^6$ | |
| | VA[b] | 88 | 34 | $3.86 \cdot 10^5$ | [58] |

Reactions were performed in citrate buffer (pH 4.5[a] or 7.0[b] / 5.5[c]) in the presence of 2 mM $H_2O_2$ at 25°C as described under 2.3 in the manuscript.
The data are means of three parallel experiments with standard deviation <5%.

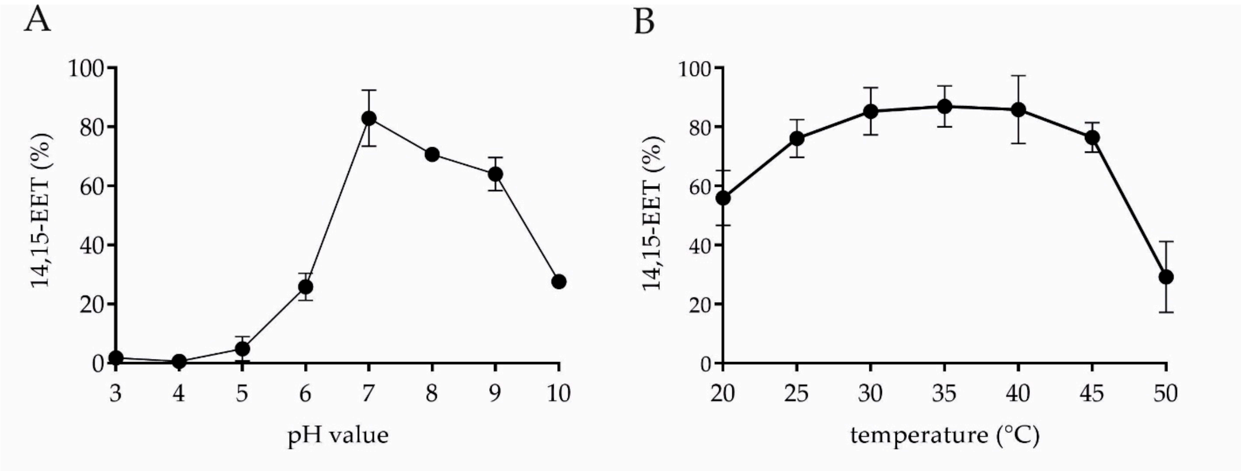


**Figure S5.** Effect of pH and temperature on the conversion of AA to 14,15-EET by *Tan*UPO. **A:** Reactions were performed in different buffers: citrate-phosphate buffer pH 3-5, sodium phosphate buffer pH 6-8, ammonium buffer pH 9-10. All buffers were used at a final concetration of 20 mM. **B:** Reactions were performed at different temperatures ranging from 20 °C to 50 °C. In all cases, conversions were performed with 2U $mL^{-1}$ *Tan*UPO, 1 mM AA, 20% (v/v) acetone and 0.04 U $mL^{-1}$ GOx for 2 h. Data are expressed as mean ± SD (n=6).

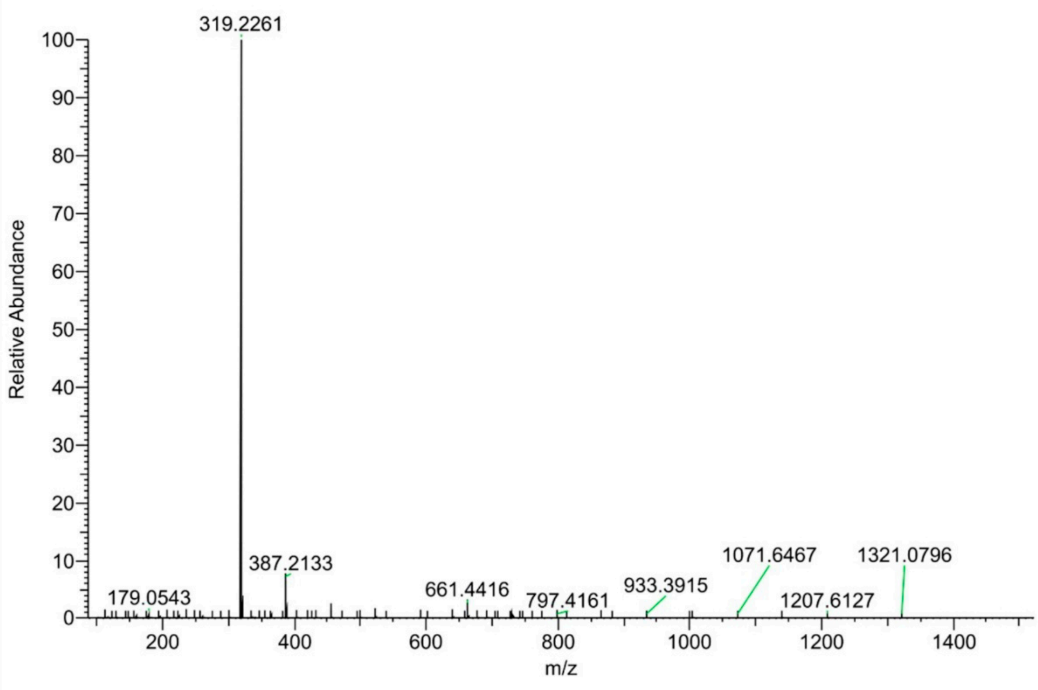


**Figure S6.** Full MS spectra of EETs and HETEs formed by UPOs. This spectrum represents each of the four main metabolites (18-HETE, 19-HETE, 11,12-EET, 14,15-EET) with an incorporation of one oxygen (+16) catalyzed by UPOs.

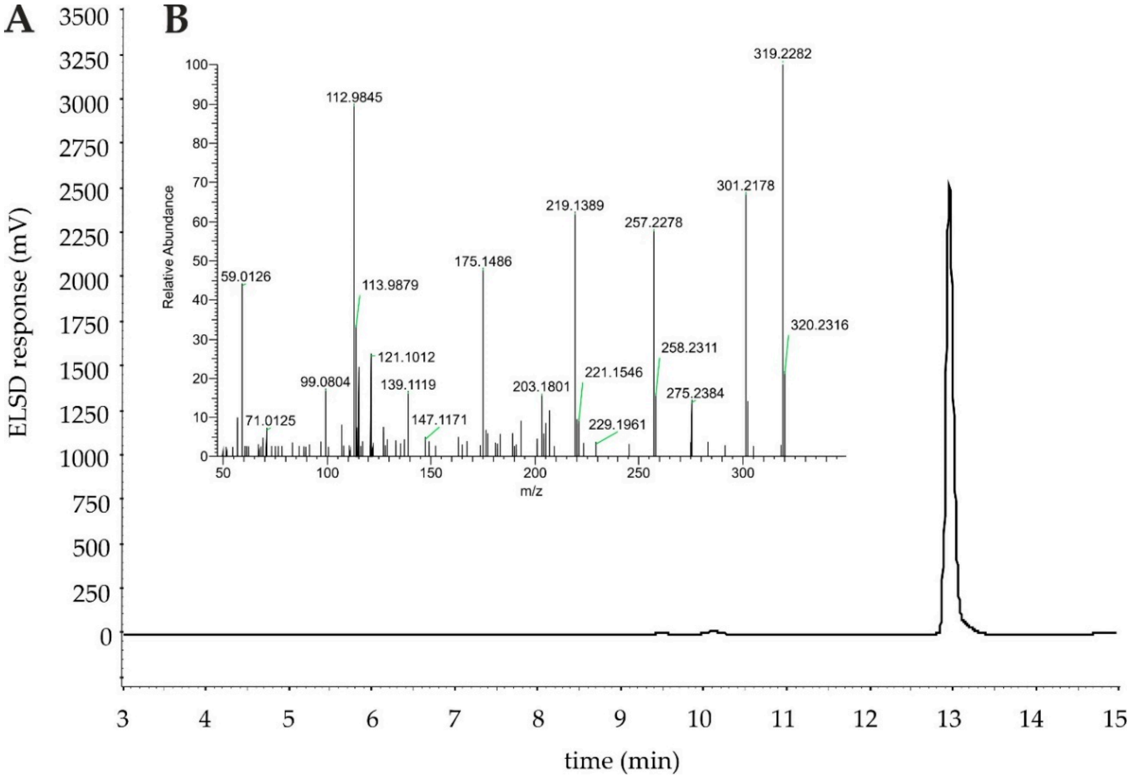


**Figure S7.** 14,15-EET reference standard. **A:** HPLC-ELSD chromatogram of 14,15-EET with RT = 13.0 min. **B:** LC-MS spectra ($MS^2$) of product ions of 14,15-EET. [M-H]- 319 product ions:, m/z =219 (loss of the neutral aldehyde with proton rearrangement), m/z = 175 (loss of $CO_2$ from previously formed fragment), m/z = 113 (an enolate anion fragment), m/z = 301 (-$H_2O$), m/z = 257 [-($H_2O$ + $CO_2$)].

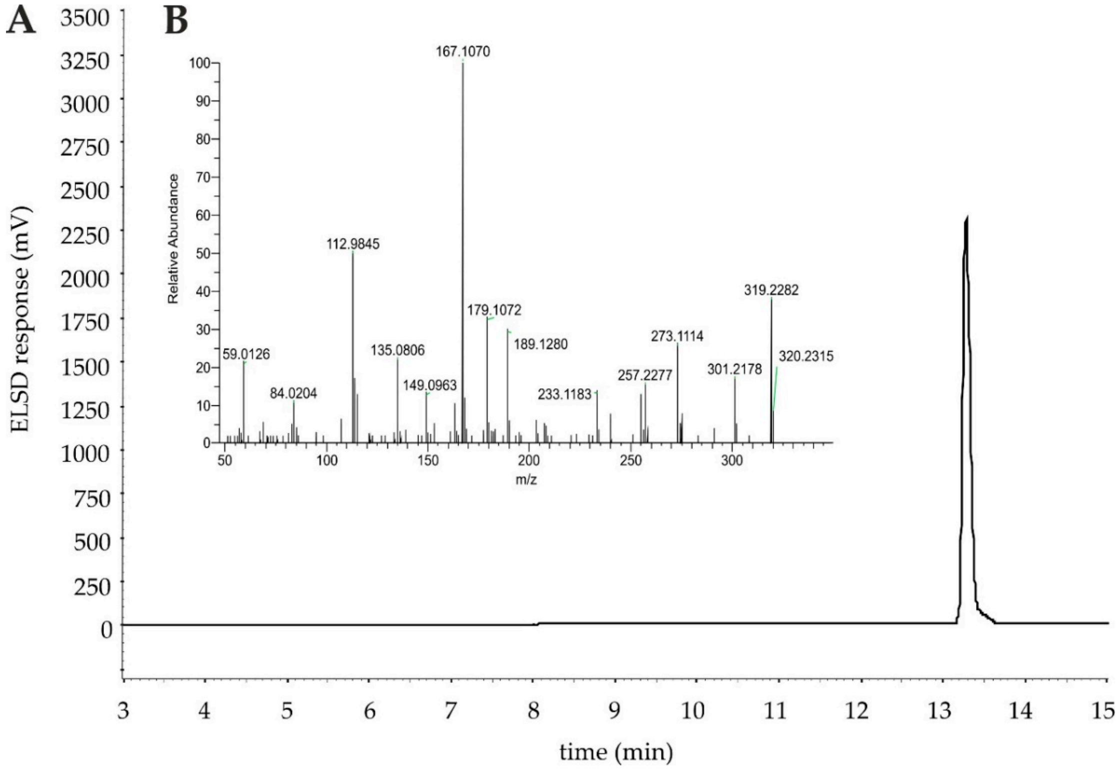


**Figure S8.** 11,12-EET reference standard. **A:** HPLC-ELSD chromatogram of 11,12-EET with RT = 13.3 min. **B:** LC-MS spectra ($MS^2$) of product ions of 11,12-EET. [M-H]- 319 product ions: m/z = 301 (-$H_2O$), m/z = 257 [- ($H_2O$ + $CO_2$)], m/z = 167, m/z = 179 and m/z = 208.

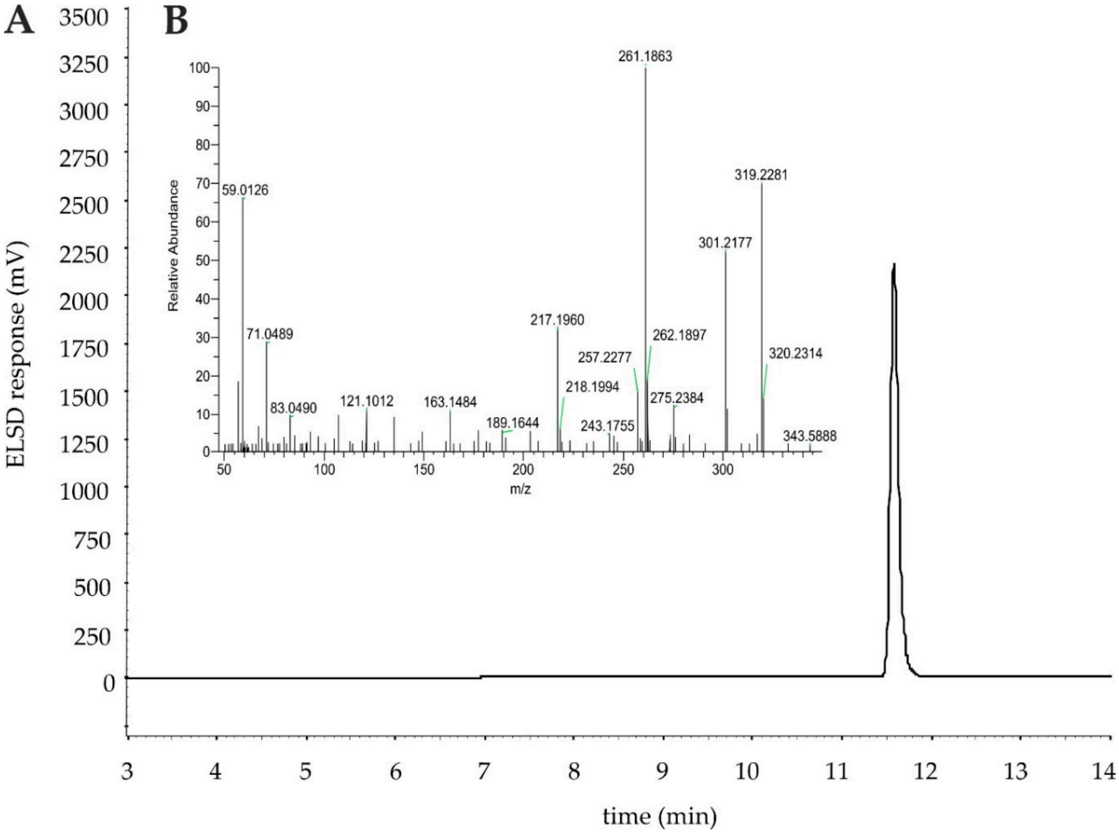


**Figure S9.** 18-HETE reference standard. A: HPLC-ELSD chromatogram of 18-HETE with RT = 11.6 min. B: LC-MS spectra (MS2) of product ions of 18-HETE. [M-H]- 319 product ions: m/z = 261, m/z = 217 (loss of $CO_2$ from previously formed fragment), m/z = 301 (-$H_2O$), m/z = 257 [-($H_2O$ + $CO_2$)].

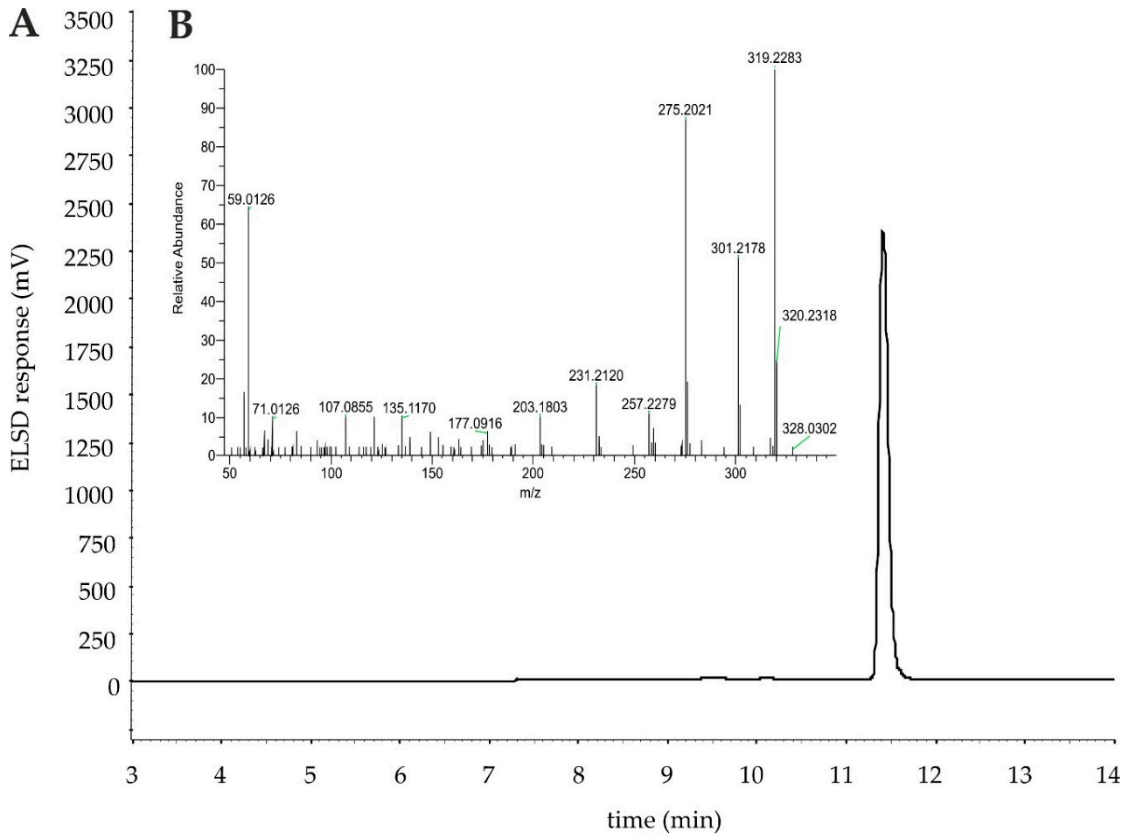


**Figure S10.** 19-HETE reference standard. A: HPLC-ELSD chromatogram of 19-HETE with RT = 11.4 min. B: LC-MS spectra (MS[2]) of product ions of 19-HETE. [M-H]- 319 product ions: m/z = 275, m/z = 231 (loss of $CO_2$ from previously formed fragment), m/z = 301 (-$H_2O$), m/z = 257 [-($H_2O$ + $CO_2$)].

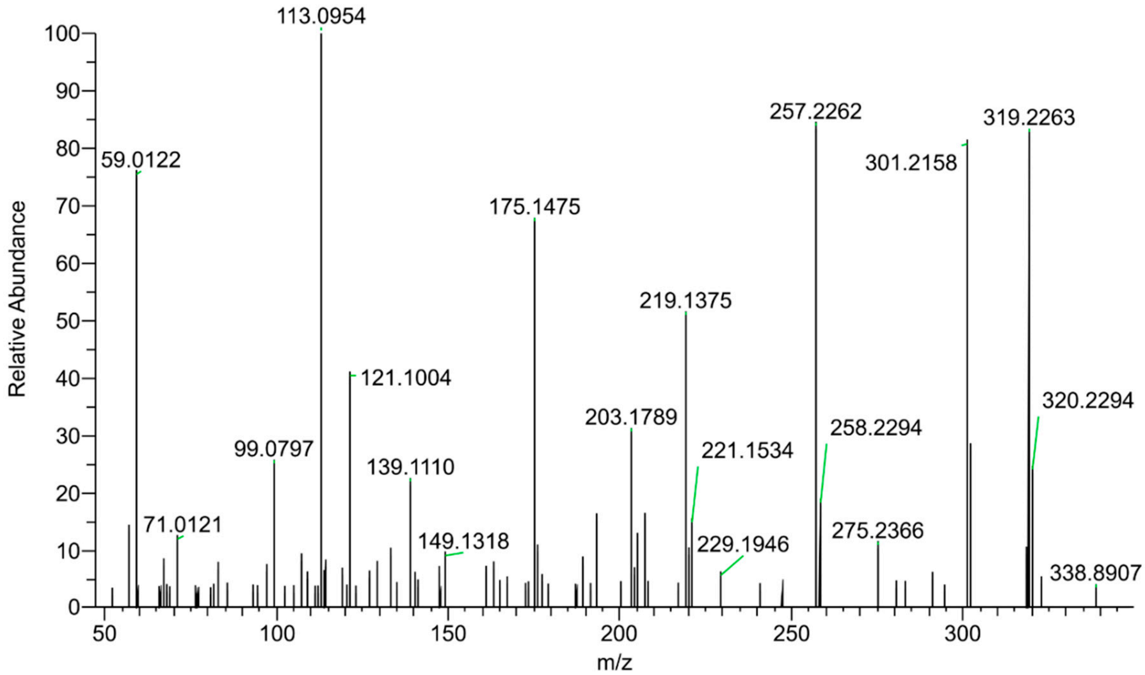


**Figure S11.** LC-MS spectra (MS[2]) of product ions of 14,15-EET formed by *Tan*UPO, *Mro*UPO, *Mwe*UPO and *Cgl*UPO. [M-H]- 319 product ions: m/z =219 (loss of the neutral aldehyde with proton rearrangement), m/z = 175 (loss of $CO_2$ from previously formed fragment), m/z = 113 (an enolate anion fragment), m/z = 301 (-$H_2O$), m/z = 257 [-($H_2O$ + $CO_2$)].

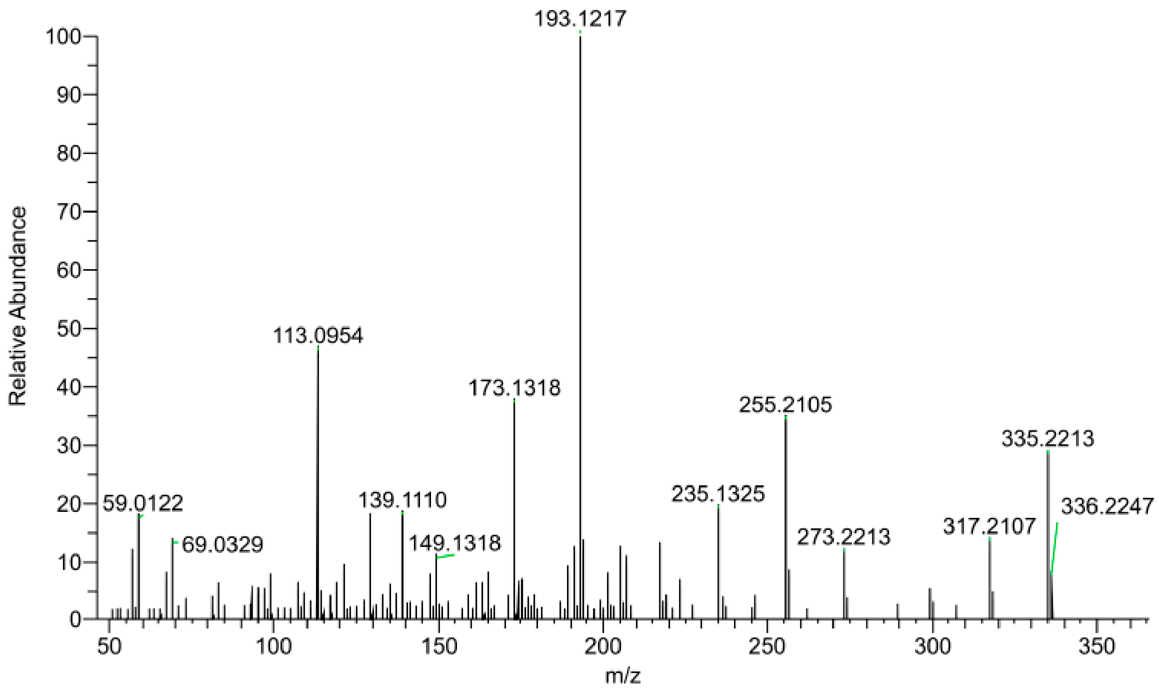


**Figure S12.** LC-MS spectra ($MS^2$) of product ions of the di-oxyfunctionalized product formed by *Tan*UPO, *Mro*UPO and *Mwe*UPO. [M-H]- 335 product ions: m/z 193, m/z 173, m/z 255, m/z 113 and m/z 235.

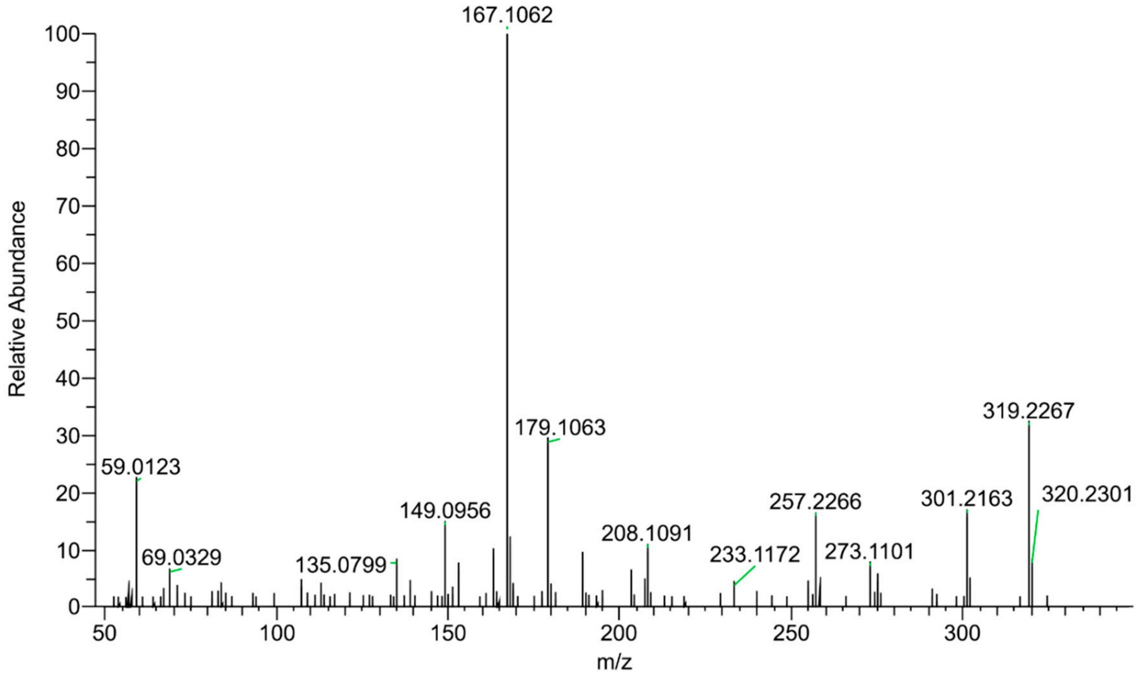


**Figure S13.** LC-MS spectra ($MS^2$) of product ions of 11,12-EET formed by *Cgl*UPO, *Mro*UPO and *Mwe*UPO. [M-H]- 319 product ions: m/z = 301 (-$H_2O$), m/z = 257 [- ($H_2O$ + $CO_2$)], m/z = 167, m/z = 179 and m/z = 208.

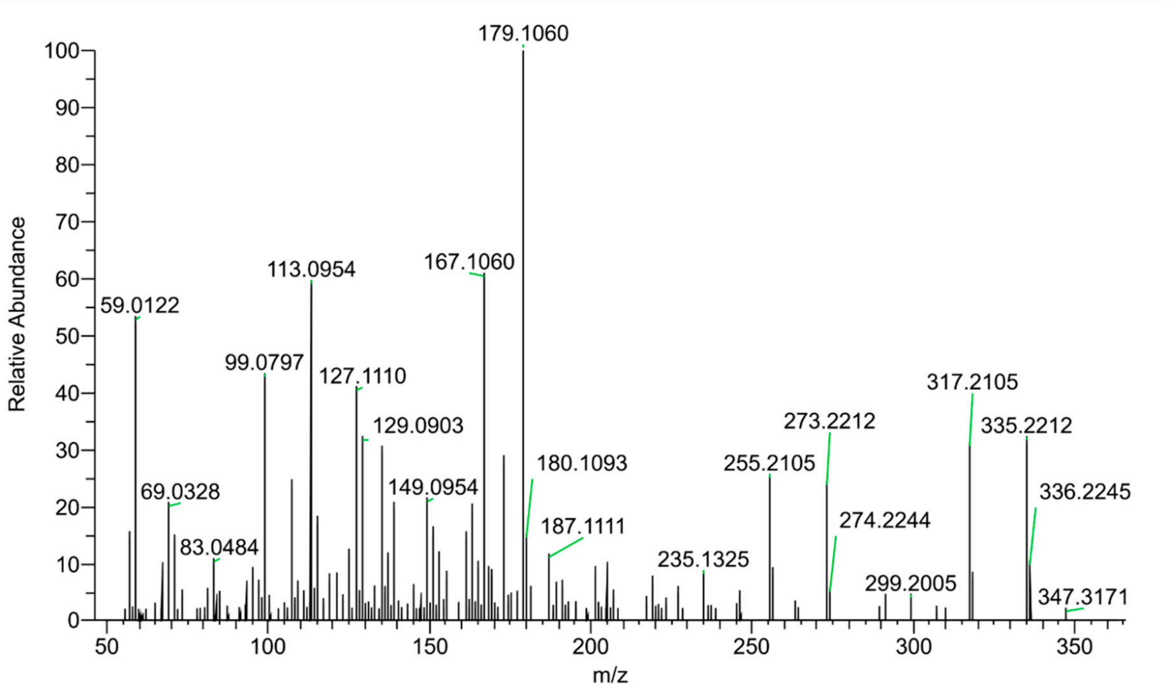


**Figure S14.** LC-MS spectra ($MS^2$) of product ions of suspected the di-epoxide formed by *Cgl*UPO. [M-H]- 335 product ions: m/z = 167, m/z = 179 and m/z = 113.

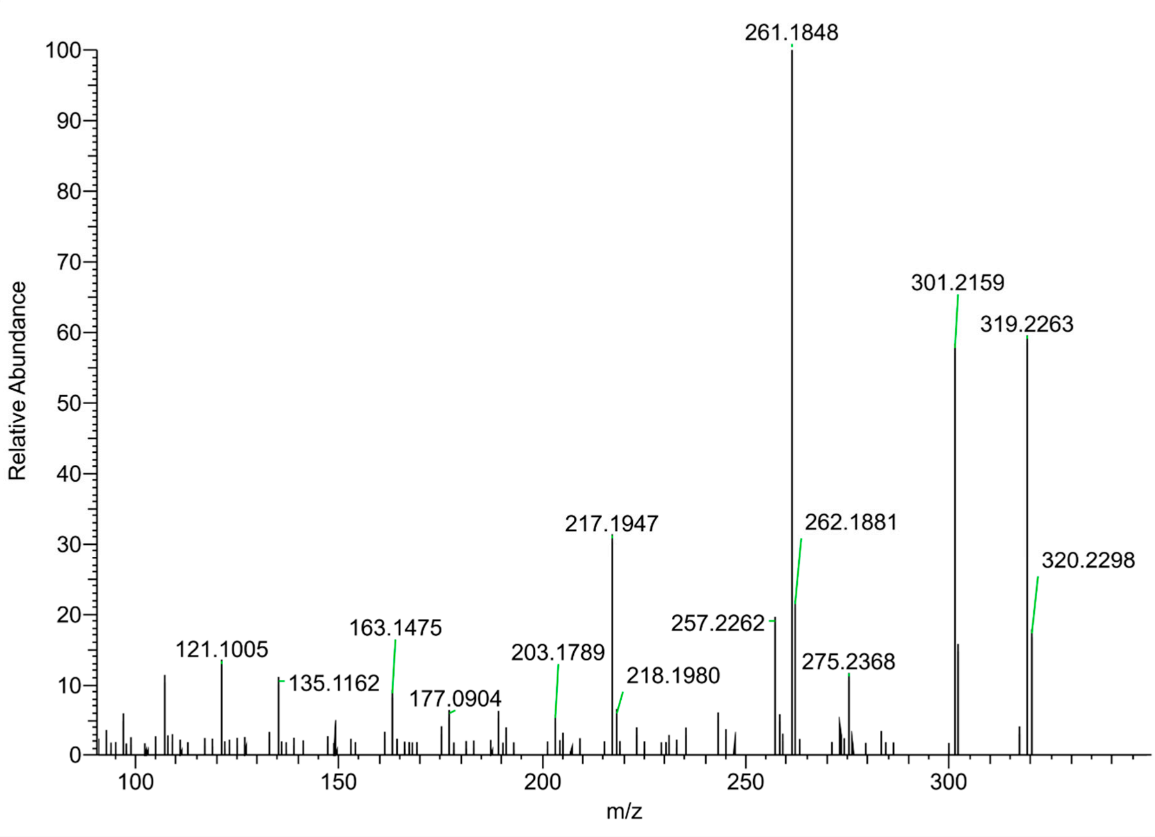


**Figure S15.** LC-MS spectra ($MS^2$) of product ions of 18-HETE formed by *Aae*UPO and *Cra*UPO. [M-H]- 319 product ions: m/z = 261, m/z = 217 (loss of $CO_2$ from previously formed fragment), m/z = 301 (-$H_2O$), m/z = 257 [-($H_2O$ + $CO_2$)].

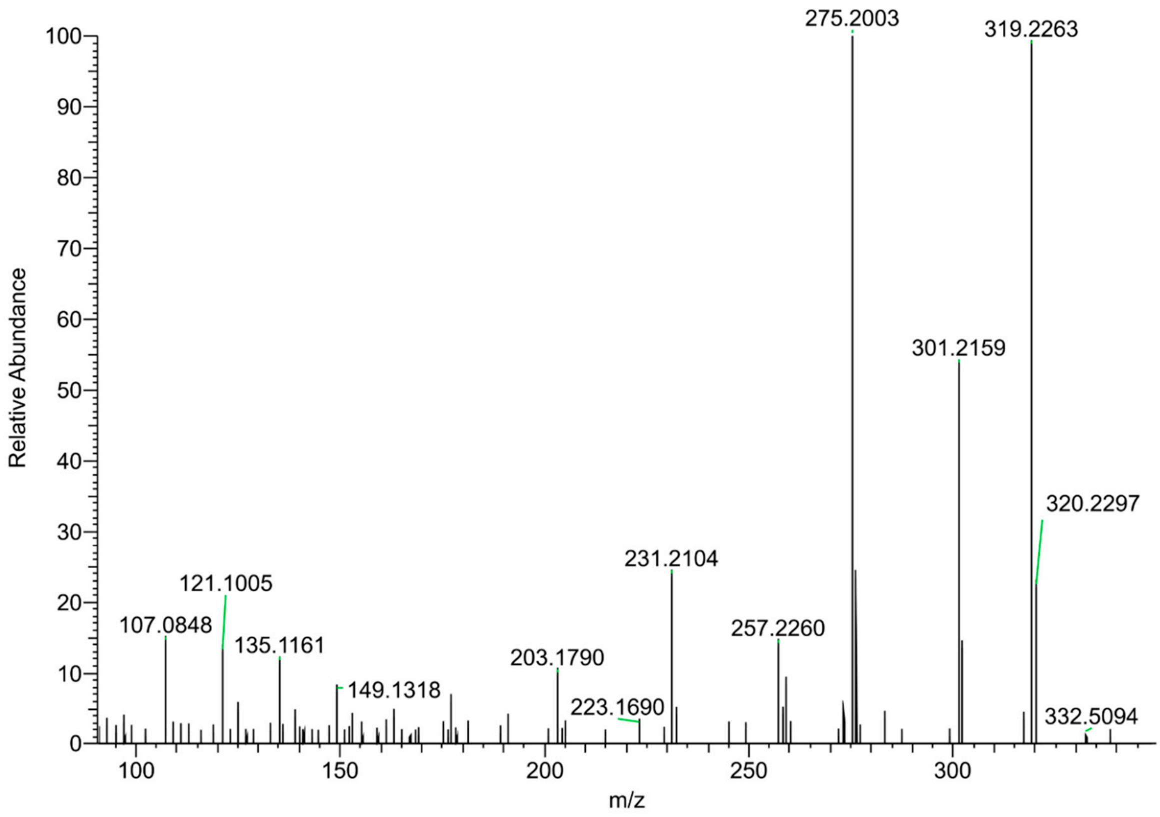


**Figure S16.** LC-MS spectra (MS[2]) of product ions of 19-HETE formed by *Aae*UPO and *Cra*UPO. [M-H]- 319 product ions: m/z = 275, m/z = 231 (loss of $CO_2$ from previously formed fragment), m/z = 301 (-$H_2O$), m/z = 257 [-($H_2O$ + $CO_2$)].

**Table S4.** $MS^1$ and $MS^2$ $[M-H]^-$ signals of by-products formed by enzymatic conversion of AA.

| Peak | MS¹ [M-H]- | MS² fragmentation [M-H]- (relative abundance %) | Structure |
|---|---|---|---|
| VI | 317.2105<br>theoret. for $C_{20}H_{30}O_3$ | 317.2106 (100),<br>57.0329 (51),<br>273.2212 ($-CO_2$)(17),<br>275.2003 (16),<br>247.1689 (13),<br>59.0122 ($-CH_3COOH$)(11),<br>259.1689 (8),<br>299.2001 ($-H_2O$)(7) | 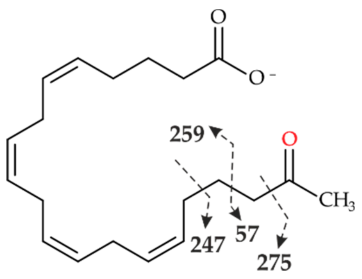 |
| VII A | 335.2216<br>theoret. for $C_{20}H_{32}O_4$ | 193.1217 (100),<br>113.0954 (52),<br>173.1318 (235 - ($CO_2$ + $H_2O$))(38),<br>255.2105 (-($2H_2O+CO_2$))(34),<br>235.1325 (20),<br>59.0122 ($-CH_3COOH$)(19),<br>317.2107 ($-H_2O$)(15),<br>273,2213 (-($H_2O+CO_2$))(12),<br>149.1318 (11) | 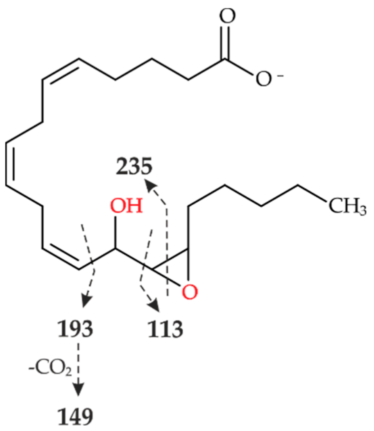 |
| VII B | 335.2211<br>theoret. for $C_{20}H_{32}O_4$ | 179.1060 (100),<br>167.1060 (56),<br>113.0954 (50),<br>127.1110 (45),<br>99.0797 (41),<br>255.2104 (-($2H_2O+CO_2$))(27),<br>273.2212 (-($H_2O+CO_2$))(21) | 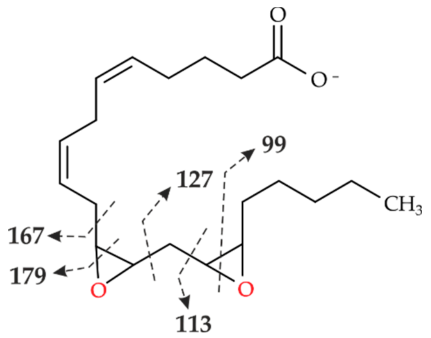 |

Peptide mapping

Each sample was prepared for proteolytic cleavage before mass-spectrometric analysis. Protein lysate was reduced (2.5 mM DTT for 1 h at 60 °C) and alkylated (10 mM iodoacetamide for 30 min at 37 °C). Proteolysis was performed overnight using trypsin (Promega, Madison, WI, USA) with an enzyme: substrate ratio of 1:25 overnight at 37°C by trypsin (Promega). Extracted peptide lysate was desalted using a C18 ZipTip column (Merck Millipore). Peptide lysates were dissolved in 0.1 % formic acid and injected into a nano liquid chromatography mass spectrometry system (nanoLC-MS/MS). Mass spectrometry was performed on a Q Exactive HF MS (Thermo Fisher Scientific, Waltham, MA, USA) with a TriVersa NanoMate (Advion, Ltd., Harlow, UK) source in the LC chip coupling mode.

First, the peptide lysates were separated on a UHPLC system (Ultimate 3000, Dionex/Thermo Fisher Scientific, Idstein, Germany). In total, 5 μL samples were loaded over 5 min on the pre-column (μ-pre-column, Acclaim PepMap, 75 μm inner diameter, 2 cm, C18, Thermo Scientific) at 4% mobile phase B (80% acetonitrile in nanopure water with 0.08% formic acid), 96% mobile phase A (nanopure water with 0.1% formic acid), then eluted from the analytical column (PepMap Acclaim C18 LC Column, 25 cm, 3 μm particle size, Thermo Scientific) over a 60-min linear gradient of mobile phase B (4-55% B). The mass spectrometer was set on loop count of 20 used for MS/MS scans with higher energy collision dissociation (HCD) at normalized collision energy of 28%. MS scans were measured at a resolution of 120,000 in the scan range of 350-1,550 m/z. MS ion count target was set to 3×106 at an injection time of 80 ms. Ions for MS/MS scans were isolated in the quadrupole with an isolation window of 1.4 Da and measured with a resolution of 15,000 in the scan range of 200-2,000 m/z. The dynamic exclusion duration was set to 30 s with a 10 ppm tolerance.

The software Proteome Discoverer (v2.5, Thermo Scientific) was used for protein identification. Therefore, the measured MS/MS spectra (*.raw files) were searched with the Sequest HT algorithm against the common contaminant protein-coding genes included in the whole genome sequence of *T. angustata* (GenBank: KAH8203164.1). The following settings were selected for both searches: enzyme specificity was trypsin with up to two missed cleavages allowed, using 10-ppm peptide ion tolerance and 0.05 Da MS/MS tolerances. Oxidation (methionine) and acetylation (any N-terminus) were selected as a dynamic modification and carbamidomethylation (cysteine) as a static modification. Only peptides with a false discovery rate (FDR) $<0.01$ calculated by Percolator and XCorr $>2.5$ were considered as identified.

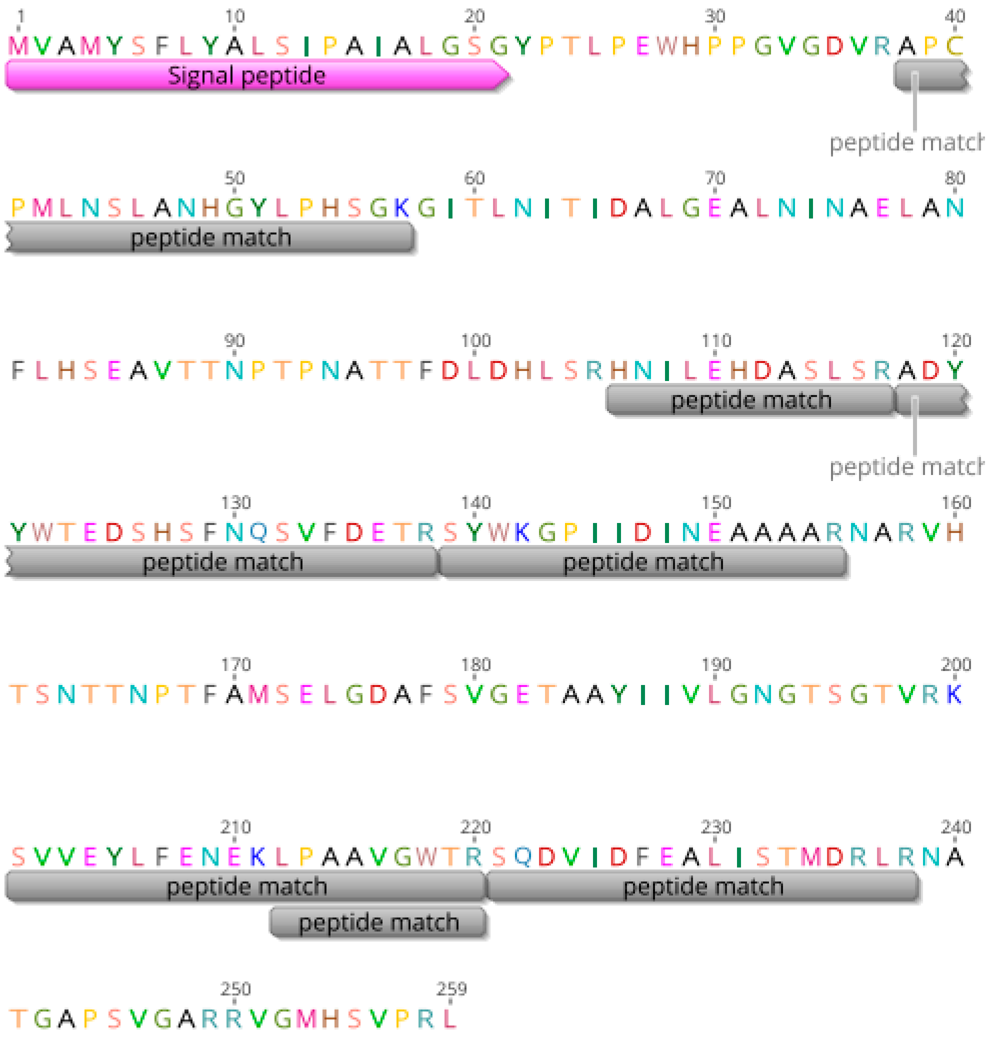


**Figure S17.** Amino acid sequence of the *Tan*UPO protein (GenBank: KAH8203164.1). The grey underlined letters represent peptides identified by petide mapping. The UPO-characteristic, highly conserved PCP-motif (proximal heme-binding site) and the EHD motif (distal binding-site for a magnesium ion) are included.

Protein-structure simulation and ligand fitting

The encoded amino acid sequence of *Tan*UPO was truncated N-terminally by 21 residues (a signal peptide) to match the mature amino acid terminus. The signal peptide was identified using SignalP6.0 [75].

The tertiary structure inference of *Tan*UPO was done using the ColabFold pipeline [82] on a local workstation (NVIDIA Quadro RTX 6000 GPU; Ubuntu 20.04.2). Multiple sequence alignments (MSA) were generated using MMSeq2 [83] searching Colabfold sequence databases. The modelling was done using AlphaFold2 [84] generating five models (3 recycle-steps each, followed by AMBER relaxation) within the LocalColabFold (https://github.com/YoshitakaMo/localcolabfold) implementation of ColabFold.

Before ligand fitting, organic molecules that occupy the substrate access channels of the pre-existing crystal structures of *Aae*UPO (PDB#: 2YOR chain A) and *Mro*UPO (PDB#: 5FUJ chain A) were removed (1H-imidazol-5-ylmethanol in *Aae*UPO, glycerol in *Mro*UPO). The heme of *Mro*UPO was transplanted into *Tan*UPO. To this end, the protein main-chain of *Mro*UPO (including positional information of its heme group) and *Tan*UPO were structurally aligned using the CEAlign algorithm [85] implemented in PyMOL (version 2.5.2).

The ligand arachidonic acid (AA) was placed into the substrate access channel of UPO structures with the module FlexAID of the plugin NRGSuite [86] (version 2.48l) in the PyMOL Molecular Graphics System (version 2.1.0 Open-Source, Schrödinger, LLC). Different AA-carbons were anchored in proximity to the heme-iron as a starting point of the procedure: C19 for *Aae*UPO, C14 for *Mro*UPO and C15 for *Tan*UPO. These were chosen to illustrate substrate channel differences across these UPOs, which may explain (retrospectively) the identified product spectra. The target structures (i.e. UPO proteins) were kept inflexible during this relatively simple simulation.

FlexAID's scoring function configuration and genetic algorithm parameters were kept at their default settings (e.g. exclude intramolecular interactions; Van der Waals permeability = 0.1; search space: angle from reference = 5.0, dihedrals from reference = 5.0, dihedrals of flexible bonds = 10.0, grid spacing = 0.375, side-chain flexibility = 0.2; genetic parameters: number of chromosomes = 500, number of generations = 500, crossover rate = 0.900, mutation rate = 0.025; shared fitness model, "population boom" reproduction model).

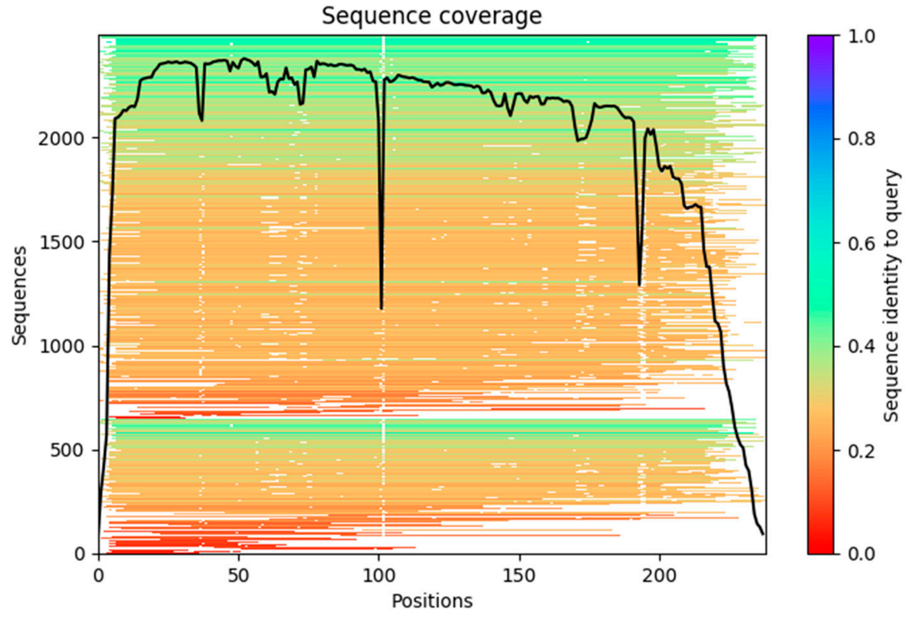


**Figure S18.** Multiple sequence alignment (MSA) plot with the assumed mature TanUPO protein sequence (i.e. signal peptide removed) as query. Alignment coverage as black line. The rainbow color-scheme represents amino acid identity, from red = lowest to violet = query-identical, to visualize alignment diversity. This is a standard output-plot generated by the ColabFold pipeline.

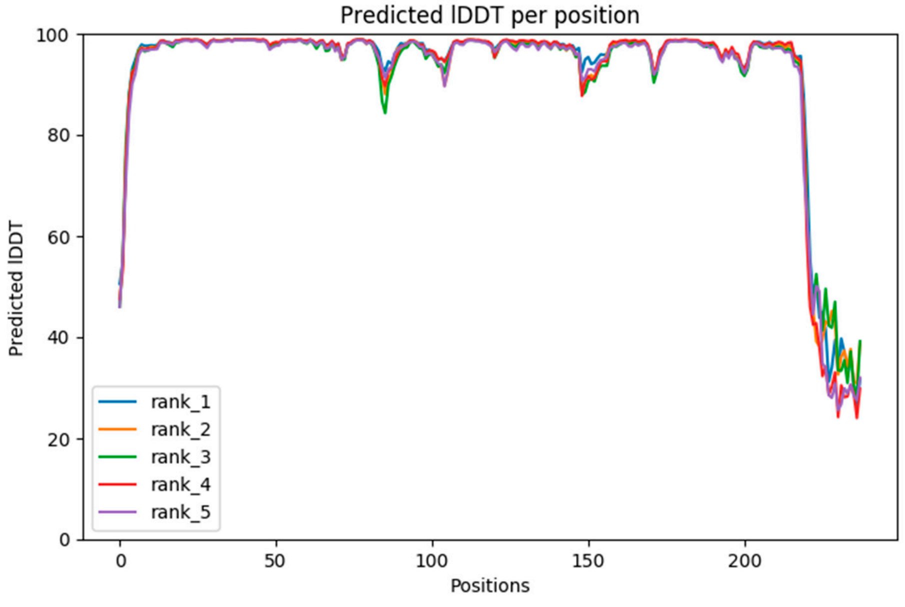


**Figure S19.** pLDDT (predicted local distance difference test) plot for the top 5 ranked TanUPO-output models of ColabFold. This plot is generated by AlphaFold2 as a per-position prediction-confidence measure. Generally, the higher the pLDDT the better the confidence in the predicted structural resolution of the model. pLDDT ≥ 80 is deemed as reliable for further structure-based analysis (as per current AlphaFold2 recommendations). All ranked models of TanUPO show low confidence (pLDDT < 80) for the first few N-terminal amino acids and 15 C-terminal amino acids (C-tail). See as well Fig. S 20.

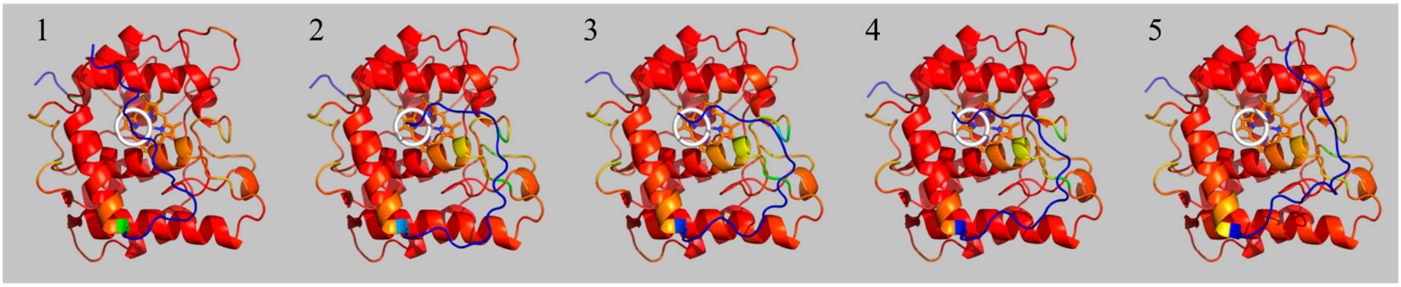


**Figure S20.** Cartoon representation of predicted *Tan*UPO models (left to right) from rank 1 to rank 5. The protein chain is colored in a rainbow-gradient, according to the pLDDT value at each position (pLDDT > 80 in red to green, pLDDT ≤ 80 in blue; pLDDT values as in Fig. S 6). A white ring is positioned at the entry to the substrate access channel; the heme is shown as sticks. This plot is meant to support the pLDDT plot in Fig. S 7 by showing the respective 3D cartoon of each model.

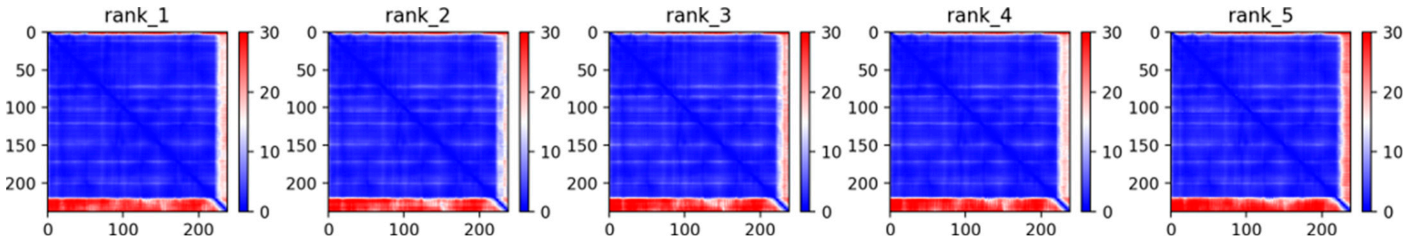


**Figure S21.** PAE (predicted alignment error) plots of the models of TanUPO. This plot helps to interpret uncertainties of the relative positioning of different domains/regions within the same protein by giving an expected position error for all pairs of residues (X, Y) at residue X. It is a standard prediction-confidence measure of AlphaFold2. A blue-white-red spectrum indicates low (in blue) to high (in red) PAEs. In general, the PAE lowers with structural order (e.g. secondary structures) and the amount of predicted inter-residue contacts between protein domains. These plots show great positional variability in particular in the 3D positioning of the C-tail (last 15 amino acids) relative to most of the remaining folded protein amino acid residues.

```
AaeUPO     LPPGPLENSSAKLVNDEAHPWKPLRPGDIRGPCPGLNTLASHGYLPRN-GVATPVQIINAVQEGLNFDNQ     69
MroUPO     ----------------SAHPWKAPGPNDSRGPCPGLNTLANHGFLPRNGRNISVPMIVKAGFEGYNVQSD     54
TanUPO     YP--------------TLPEWHPPGVGDVRAPCPMLNSLANHGYLPHSGKGITLNITIDALGEALNINAE     56

AaeUPO     AAVFATYAAH--LVDGNLITDLLSIGRKTRLTGPDPPPPASVGGLNEHGTFEGDASMTRGDAF-FGNNHD    136
MroUPO     ILILAGKIGM--L--TSREADTISLEDLKLH-----------------GTIEHDASLSREDVA-IGDNLH    102
TanUPO     LANFLHSEAVTTN--PTPNATTFDLDHLSRH-----------------NILEHDASLSRADYYWTEDSHS    107

AaeUPO     FNETLFEQLVDYSNRFGGGKYNLTVAGELRFKRIQDSIATNPNFSFVDFRFFTAYGETTFPANLFVDGRR    206
MroUPO     FNEAIFTTLAN--SNPGADVYNISSAAQVQHDRLADSLARNPNVTNTDLTATIRSSESAFFLTVMSAGDP    170
TanUPO     FNQSVFDETRS--YWKGPII-DINEAAAARNARVHTSNTTNPTFAMSELGDAFSVGETAAYIIVLGNGT-    173

AaeUPO     DDGQLDMDAARSFFQFSRMPDDF--FRAPSPRS----GTGVEVVIQAHP-MQPGRNVGKINSYTVDPTS-    268
MroUPO     LRGEAPKKFVNVFFREERMPIKEGWKRSTTPITIPLLGPIIERITELSD-WKPT-----------GDNC-    227
TanUPO     -SGTVRKSVVEYLFENEKLPAAVGWTRSQDVIDFEALISTMDRLRNATGAPSVG-----------ARRVG    231

AaeUPO     -------SDFSTPCLMYEKFVNITVKSLYPNPTVQLRKALNTNLDFFFQGVAAGCTQVFPYGRD          325
MroUPO     -------GAIVLSPEL------------------------------------------------          236
TanUPO     MHSVPRL---------------------------------------------------------          238
```

**Figure S22.** Structural alignment of *Aae*UPO, *Mro*UPO and *Tan*UPO using the CEAlign [85] algorithm as implemented in Pymol version 2.5.2 (residues aligned according to occupancy of same relative position in the folded protein). Colored underlines indicate main-chain segments depicted in Fig. 4. Side-chain residues that face the substrate access channel are colored in red letters. Text background color according to evolutionary divergence (Blosum45 matrix, black = > 80% similar, grey = 80 – 60% similar).

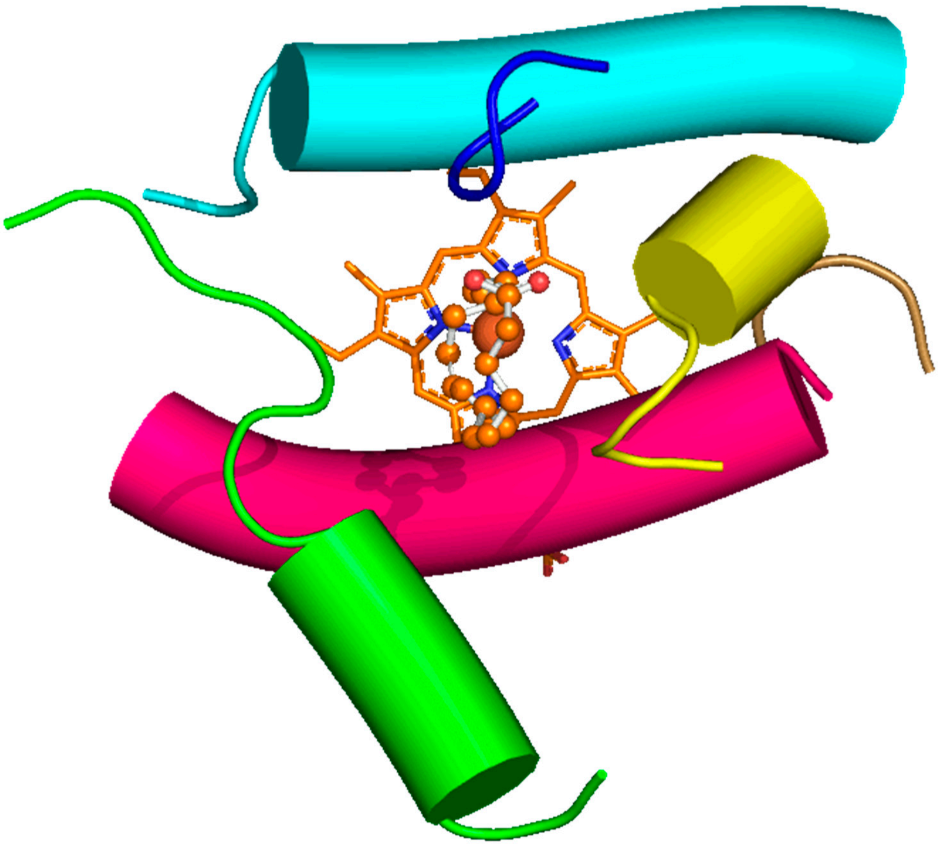

**Figure S23.** Topology of the SAC (substrate access channel) of *Aae*UPO forming helices and loops (GIF). Only the protein main-chain is depicted. Protein chains colored as in Fig. 4: helix a (Ha) = cyan, Hb = pink, Hc = green, loop a (La) = sand. The extra loops and helix unique to *Aae*UPO are depicted in blue and yellow. Helices are depicted as cylinders. The heme bonds are depicted as sticks and the heme-iron as a brown ball, AA is depicted as smaller orange balls (carbon atoms) and sticks as in Fig. 4. The first frame is a top-down view into the SAC.

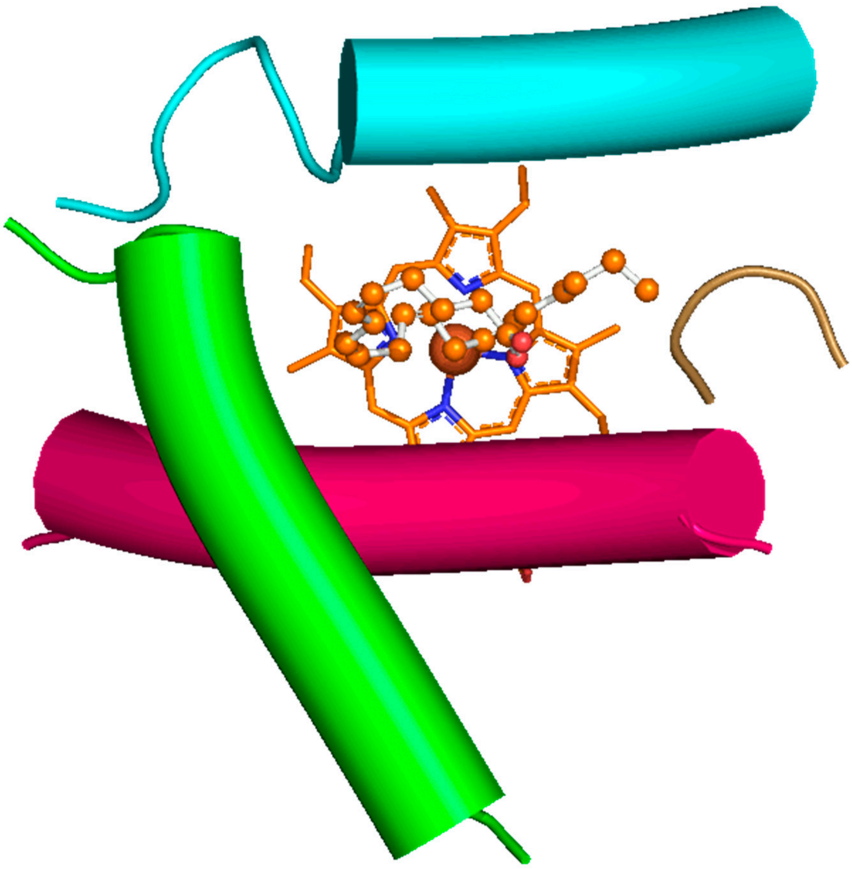

**Figure S24.** Topology of the SAC (substrate access channel) of *Mro*UPO forming helices and loops (GIF). Only the protein main-chain is depicted. Protein chains colored as in Fig. 4: helix a (Ha) = cyan, Hb = pink, Hc = green, loop a (La) = sand. Helices are depicted as cylinders. The heme bonds are depicted as sticks and the heme-iron as a brown ball, AA is depicted as smaller orange balls (carbon atoms) and sticks as in Fig. 4. The first frame is a top-down view into the SAC.

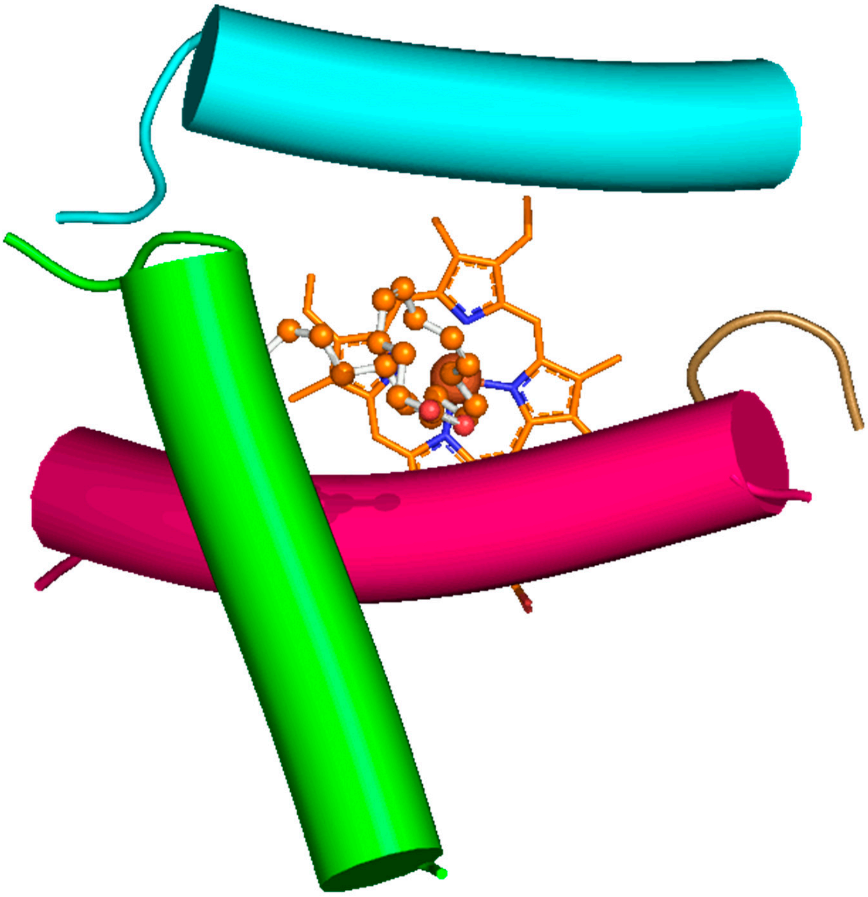

**Figure S25.** Topology of the SAC (substrate access channel) of *Tan*UPO forming helices and loops (GIF). Only the protein main-chain is depicted. Protein chains colored as in Fig. 4: helix a (Ha) = cyan, Hb = pink, Hc = green, loop a (La) = sand. Helices are depicted as cylinders. The heme bonds are depicted as sticks and the heme-iron as a brown ball, AA is depicted as smaller orange balls (carbon atoms) and sticks as in Fig. 4. The first frame is a top-down view into the SAC.

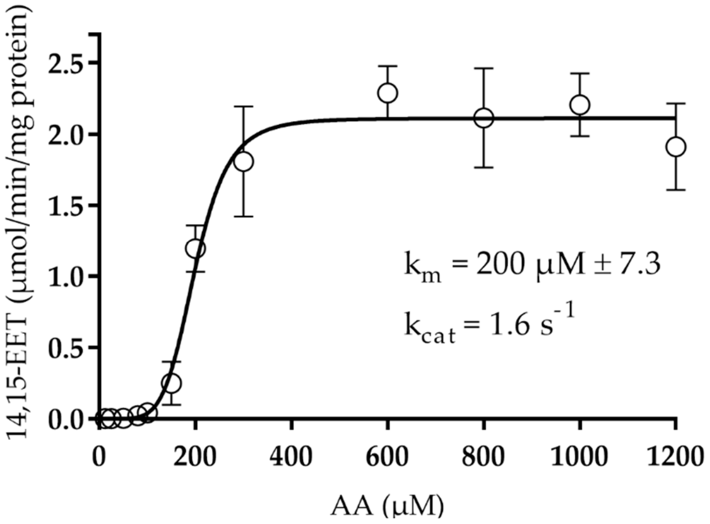


**Figure S26.** Kinetic profile of arachidonic acid (AA) conversion to 14,15-EET by *Tan*UPO. Reactions were performed in sodium phosphate buffer (pH 7.0) in the presence of 0.5 mM $H_2O_2$ at 30°C for 3 min. The concentrations of the AA metabolite 14,15-EET were calculated by comparing the corresponding analyte/reference response ratio based on the peak area to calibration curve in the $MS^1$ spectrum of LC-MS/MS. Substrate concentration was varied between 12.5 and 1,200 μM, and 20% (v/v) acetone was used as cosolvent. The time course of the reactions was previously analyzed to ensure that the kinetic parameters were calculated when the reaction rate was still in the linear phase. The experimental values for arachidonic acid metabolism were expressed as mean± SD (n = 6).
