# Supplementary figures and images for: Novel Unspecific Peroxygenase from Truncatella angustata Catalyzes the Synthesis of Bioactive Lipid Mediators
Source: Microorganisms. 2022 Jun 22;10(7):1267. doi: 10.3390/microorganisms10071267 (PMC9322767; doi:10.3390/microorganisms10071267)

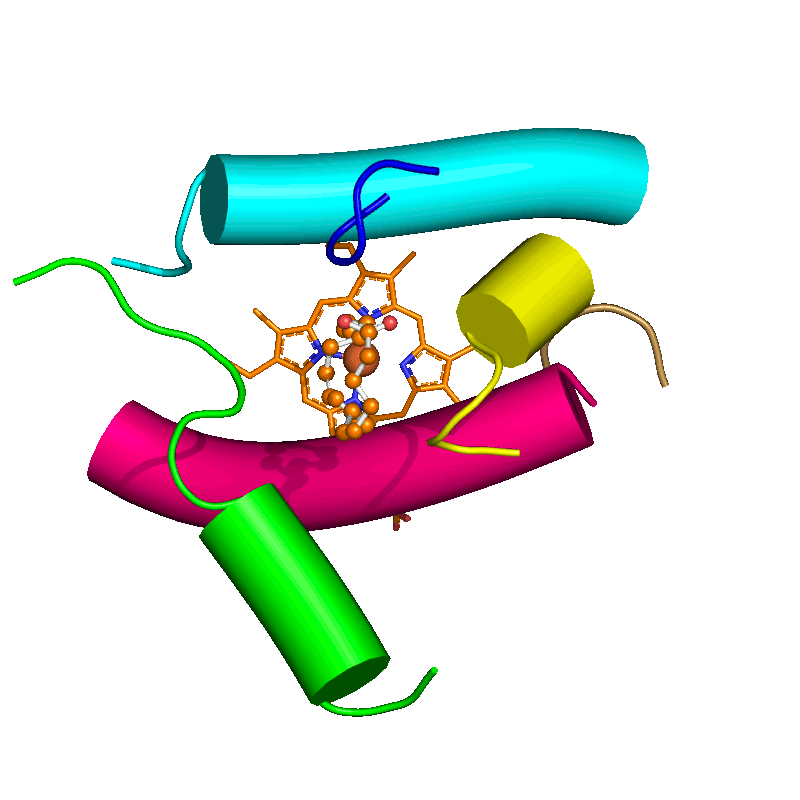

Supplement: Supplementary file 1 [file microorganisms-10-01267-s001.zip › Figure S23.gif]

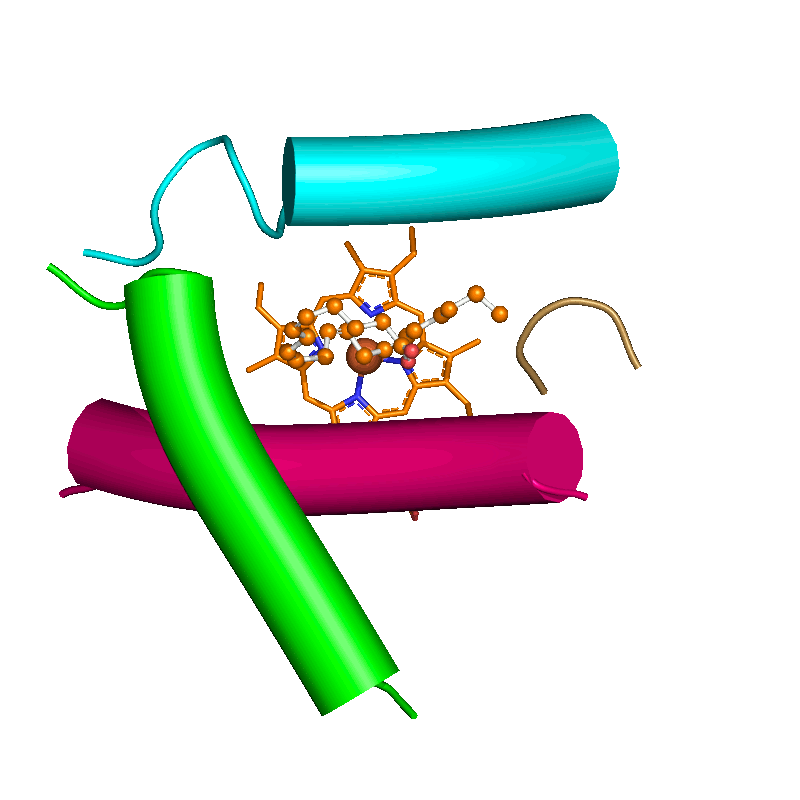

Supplement: Supplementary file 1 [file microorganisms-10-01267-s001.zip › Figure S24.gif]

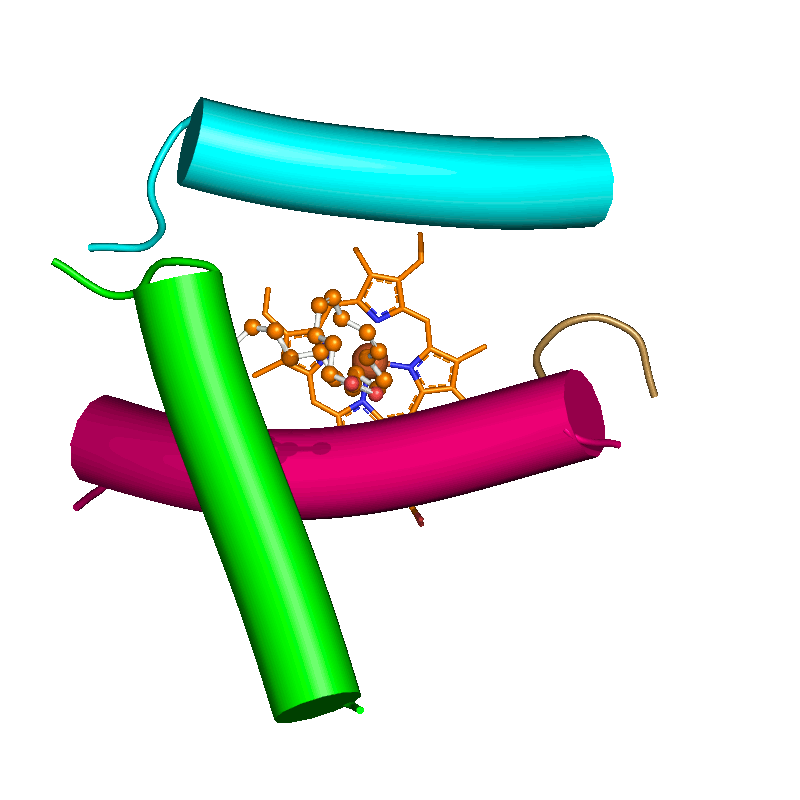

Supplement: Supplementary file 1 [file microorganisms-10-01267-s001.zip › Figure S25.gif]
